# Juglone/MWCNT-Modified Electrode for High-Performance Melatonin Detection

**DOI:** 10.3390/ijms27146237

**Published:** 2026-07-13

**Authors:** Joanna Smajdor-Baran

**Affiliations:** Department of Analytical Chemistry and Biochemistry, Faculty of Materials Science and Ceramics, AGH University of Krakow, 30-059 Krakow, Poland; smajdorj@agh.edu.pl

**Keywords:** juglone, MWCNTs, voltammetry, melatonin, paste electrode

## Abstract

The integration of carbon nanomaterials with organic compounds offers a promising strategy for developing next-generation electrode materials with superior properties. A novel type of carbon paste electrode was fabricated by modifying a graphite matrix with functionalized multiwalled carbon nanotubes and juglone as a redox mediator, and then it was deposited by manual packing into a PEEK body (JUG-MWCNT/CPE). The surface morphology and structural parameters of the composite materials were meticulously characterized using scanning electron microscopy (SEM), nitrogen adsorption–desorption isotherms, and spectroscopic techniques, while their electrochemical properties were rigorously evaluated using cyclic voltammetry (CV) and differential pulse voltammetry (DPV). This study demonstrates that the synergistic interaction between the conductive nanotube network and the electroactive juglone significantly reduces the oxidation overpotential and enhances the peak current response of melatonin. Under optimized DPV parameters, the developed sensor presents outstanding analytical performance, featuring a wide linear response range from 0.002 to 0.16 mg L^−1^, a low detection limit of 0.49 µg L^−1^, excellent long-term signal stability for up to 30 days, and valid applicability for real-sample monitoring in commercial tablets and dietary supplements.

## 1. Introduction

Melatonin (N-acetyl-5-methoxytryptamine) is a vital neurohormone, primarily secreted by the pineal gland, playing a fundamental role in regulating the circadian rhythm; sleep–wake cycles; and various physiological processes, including immune responses and antioxidant defense. As a potent free radical scavenger, melatonin protects cellular membranes and DNA from oxidative stress, making it a crucial factor in cytoprotection [1,2,3,4,5,6]. Due to its significant impact on human health, fluctuations in melatonin levels are often linked to sleep disorders, depression, seasonal affective disorder, and neurodegenerative diseases such as Alzheimer’s and Parkinson’s disease [7,8,9,10,11,12,13,14,15,16]. Consequently, the development of rapid, sensitive, and reliable analytical methods for the determination of melatonin in pharmaceutical formulations and complex biological matrices is of paramount importance in clinical diagnostics and pharmacological research.

Among various analytical techniques, such as high-performance liquid chromatography (HPLC) [17,18,19,20,21], capillary electrophoresis [22,23], and enzyme-linked immunosorbent assay (ELISA) [24,25,26], electrochemical methods have garnered substantial attention. These techniques offer distinct advantages, including high sensitivity, cost-effectiveness, ease of operation, and the potential for on-site miniaturization. However, the electrochemical detection of melatonin at traditional unmodified electrodes (e.g., glassy carbon or graphite) is often hindered by high overpotentials and the fouling of the electrode surface caused by the adsorption of oxidation products [27,28,29]. These limitations result in poor reproducibility and sluggish electron transfer kinetics.

To overcome these challenges, surface modification of electrodes with advanced nanomaterials and redox-active mediators has become a prevalent strategy [30,31,32,33,34]. In recent years, multiwalled carbon nanotubes (MWCNTs) have been extensively utilized in sensor fabrication due to their exceptional electrical conductivity, high surface-to-volume ratio, and remarkable electrocatalytic properties. MWCNTs act as molecular wires, facilitating electron transfer between the analyte and the electrode substrate. Furthermore, the functionalization of MWCNTs, typically through oxidative acid treatment, introduces oxygen-containing moieties (such as carboxyl and hydroxyl groups) that enhance dispersibility and provide active sites for synergistic interaction with other modifiers via π-π stacking or electrostatic forces [35,36,37,38].

A promising direction in the design of hybrid composite sensors involves the incorporation of natural bioactive compounds as mediators. Juglone (5-hydroxy-1,4-naphthalenedione), a naturally occurring phenolic compound found in the Juglandaceae family, exhibits reversible and stable redox behavior. Its naphthoquinone backbone allows it to act as an effective electron transfer mediator, bridging the energy gap for the oxidation of organic molecules. When integrated into a carbon paste matrix, juglone can facilitate proton-coupled electron transfer mechanisms, often involved in the oxidation of indole-derivative compounds like melatonin [39,40,41,42,43]. The integration of juglone into a carbon paste electrode (CPE) matrix, alongside functionalized MWCNTs, creates a unique hierarchical architecture. This combination aims to exploit the high effective surface area of the nanotubes and the catalytic mediation of juglone to lower the activation energy of the melatonin oxidation process.

In this work, we report for the first time the development and optimization of a novel carbon paste electrode modified with functionalized MWCNTs and juglone (JUG-MWCNT/CPE) for the highly sensitive voltammetric determination of melatonin. The synergistic effect of these components allows for a significant reduction in oxidation overpotential and a substantial enhancement in the peak current response compared to bare or mono-modified electrodes. The analytical performance of the proposed sensor is rigorously evaluated using cyclic voltammetry (CV) and differential pulse voltammetry (DPV), focusing on sensitivity, selectivity in the presence of common interferents, and long-term stability for practical applications in real-sample analysis.

## 2. Results

### 2.1. Characterization of the Developed Sensors and Used Materials

#### Characterization of Electrode Modifiers

**Figure 1:**
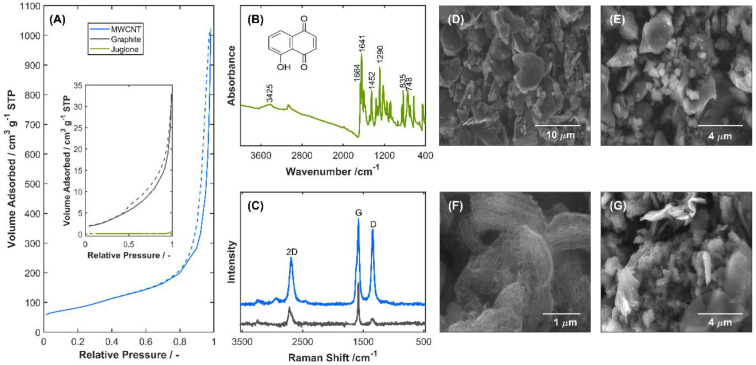
(**A**) The nitrogen adsorption–desorption isotherms of MWCNTs, graphite, and juglone (insert). Solid lines represent adsorption, whereas dashed ones represent desorption. (**B**) FT-IR spectra of juglone in the middle infrared region. (**C**) Raman spectrum of MWCNT and graphite in the range of 500–3500 cm^−1^. SEM image of (**D**) graphite, (**E**) juglone, (**F**) MWCNT, and (**G**) JUG-MWCNT–graphite composite mixture.

Figure 1A shows N_2_ adsorption–desorption isotherms of the employed MWCNT, graphite, and juglone. The shape of the isotherm of MWCNT, together with the evident adsorption–desorption hysteresis loop at high relative pressures, is characteristic of a type IV isotherm according to the IUPAC classification [44], indicating the presence of mesoporous structures formed by the entangled nanotube network. In contrast, graphite shows significantly lower nitrogen uptake, with the adsorbed volume remaining below 35 cm^3^ g^−1^ STP. The juglone sample exhibits negligible nitrogen adsorption over the entire pressure range, with only a very slight increase close to saturation pressure. This behavior indicates a non-porous character with a minimal access surface area for N_2_ adsorption. The textural parameters calculated from the nitrogen adsorption–desorption measurements are summarized in Table 1. Among the investigated materials, MWCNT exhibited the highest specific surface area, with an SBET value of 290.56 m^2^ g^−1^, which is substantially higher than that obtained for graphite and juglone. The large external surface area of MWCNT, together with the presence of a measurable micropore volume, suggests the coexistence of both micro- and mesopores within the carbon nanotube assembly. In contrast, graphite and juglone showed negligible microporosity, which agrees with their limited nitrogen uptake. The particularly small average pore diameter observed for juglone suggests a predominantly compact, non-porous structure with very limited accessibility for nitrogen molecules.

The FT-IR spectrum of juglone (Figure 1B) confirms the presence of characteristic functional groups associated with 5-hydroxy-1,4-naphthoquinone. A broad band observed at approximately 3425 cm^−1^ is attributed to the stretching vibrations of the hydroxyl (–OH) group involved in intermolecular hydrogen bonding [45]. The intense absorption bands at 1664 cm^−1^ and 1641 cm^−1^ correspond to the stretching vibrations of conjugated carbonyl groups (C=O) characteristic of the quinone structure. The band located at 1452 cm^−1^ can be assigned to aromatic stretching vibrations (C=C) within the naphthalene ring system. The strong signal at 1290 cm^−1^ is related to stretching vibrations of the phenolic group (C–O). Additionally, the absorption bands observed at 835 cm^−1^ and 748 cm^−1^ are associated with out-of-plane bending vibrations of aromatic (C–H) bonds, confirming the substituted aromatic structure of juglone [46].

To obtain structural information about graphite and MWCNT, Raman spectroscopy measurement was employed (Figure 1C). Both materials exhibit the characteristic carbon-related D, G, and 2D bands, confirming their graphitic structure. The G band, observed at approximately 1580 cm^−1^, is associated with the in-plane stretching vibration of sp^2^-hybridized carbon atoms and reflects the degree of graphitic ordering. In contrast, the D band located near 1350 cm^−1^ originates from structural defects, lattice distortions, and disorder within the carbon framework [47]. In the graphite spectrum, the sharp and intense G band, accompanied by a relatively weak D band, indicates a highly ordered crystalline graphitic structure with a low concentration of defects. Compared to graphite, the MWCNT sample exhibits a significantly higher intensity of the D band relative to the G band, indicating a higher defect density and lower structural order resulting from the curved nanotube morphology. The broader and more intense 2D band around 2650 cm^−1^ observed for MWCNT further confirms the multilayered graphitic nature of the nanotubes.

SEM images of the individual materials and their composite mixture are presented in Figure 1D–G. From a morphological point of view, graphite (Figure 1D) exhibits a characteristic layered, platelet-like structure composed of irregular flakes several micrometers in lateral size. The particles possess relatively smooth surfaces and stacked lamellar structures, characteristic of graphitic carbon materials. Agglomeration of the flakes is also visible as a result of strong van der Waals interactions between graphite layers. Figure 1E shows the morphology of juglone particles, which appear as irregular crystalline aggregates with smaller particle sizes than graphite. Juglone crystals display fragmented and angular features distributed between larger particles, indicating a heterogeneous microstructure. The SEM image of MWCNTs (Figure 1F) reveals an entangled network of multiwalled carbon nanotubes forming a porous, web-like architecture. The nanotubes are highly intertwined and randomly orientated, generating a continuous conductive network with a large surface area. In Figure 1G, representing the mixture of graphite, juglone, and MWCNT, all three morphologies can be identified simultaneously. Large graphite flakes are embedded within a matrix composed of juglone particles and interconnected MWCNT bundles. The nanotubes appear to bridge adjacent particles and graphite sheets, improving interparticle contact and producing a more compact and interconnected composite structure. This heterogeneous arrangement suggests enhanced conductive pathways and increased interfacial interactions between the composite components. The simultaneous identification of graphite flakes, juglone crystals, and interwoven MWCNT bundles in the composite matrix highlights a cohesive, heterogeneous microarchitecture. The nanotubes successfully bridge adjacent graphite and juglone particles, creating a compact network with maximized interfacial interaction. Driven by the strong affinity and non-covalent π–π stacking between the aromatic rings of juglone and the graphene walls of the MWCNTs, this structural alignment provides exceptional chemical stability and prevents modifier leaching. This structural synergy directly governs the sensor’s high electroactive surface area and facilitated charge transport.

### 2.2. Electrochemical Features of Fabricated Sensors

**Figure 2:**
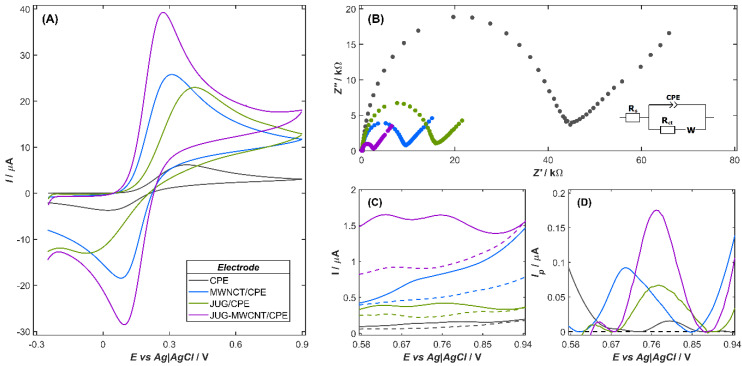
(**A**) CV curves recorded for the 2 mmol L^−1^ redox probe of [Fe(CN)_6_]^4−^ in 0.1 mol L^−1^ KCl solution (scan rate *v* = 0.05 V s^−1^) on CPE, MWCNT/CPE, JUG/CPE, and JUG-MWCNT, and (**B**) corresponding impedance spectra in the form of a Nyquist plot (inset: Randles cell equivalent circuit). Comparison of DP voltammograms obtained for 0.2 mg L^−1^ MEL in citrate buffer solution (pH 3.0) on CPE, MWCNT/CPE, JUG/CPE, and JUG-MWCNT (**C**) before and (**D**) after background subtraction step. Dashed lines represent the background signal. DPV experimental conditions: *E_p_* = 0.4 V, *E_k_* = 1.1 V, *E_s_* = 5 mV, *dE* = 50 mV, *t_s_* = 10 ms, and *t_w_* = 20 ms.

The electrochemical behavior of the electrodes was investigated using CV (Figure 2A) and EIS (Figure 2B) measurements performed in a 0.1 mol L^−1^ KCl solution containing 2 mmol L^−1^ of the [Fe(CN)_6_]^4−^ model redox probe. The impedance spectra, presented as Nyquist plots, were fitted using the equivalent electrical circuit consisting of the resistance of the solution (*R_s_*), charge transfer resistance (*R_ct_*), constant phase element (*CE*), and Warburg impedance (*W*), as shown in the inset of Figure 2B. Based on the results of the fitting, the main electrochemical parameters of CPE, MWCNT/CPE, JUG/CPE, and JUG-MWCNT/CPE were calculated and are summarized in Table 2. The charge transfer resistance (*R_ct_*) and Warburg coefficient (σ) values for the spectra recorded at the formal potential (*E_f_*) of the redox probe were determined using Equations (1) and (2), respectively:

(1) Rct=RTn2F2Aks·2(DODR)14DO12·CO+DR12·CR

(2) σ=RTn2F2A2·4DO12·CO+DR12·CR

where *R* represents the universal gas constant (R = 8.314 J mol^−1^ K^−1^); *T* represents the temperature [K]; *F* represents the Faraday constant (F = 96,485.3 C mol^−1^); *n* represents the number of transferred electrons (*n* = 1); *A* represents the electroactive surface area of the electrode [mm^2^]; and *D_O_* and *D_R_* represent the diffusion coefficients of the oxidized and reduced forms (7.2·10^−6^ cm^2^ s^−1^), respectively, and *C_O_* and *C_R_* are their concentrations in the solution.

The heterogeneous electron transfer rate constant (*k_s_*) was determined from the impedance data by combining Equations (1) and (2), resulting in the expression given in Equation (3):

(3) $$k_{s}=\frac{\sigma }{R_{ct}}\cdot \sqrt{\frac{D_{O/R}}{2}}$$

where *D_O/R_* indicates the average diffusion coefficient of the oxidized and reduced forms.

The CV responses (Figure 2A) of CPE, MWCNT/CPE, JUG/CPE, and JUG–MWCNT/CPE revealed a pronounced enhancement of the electrochemical performance after electrode modification. Compared with the bare CPE, all modified electrodes exhibited significantly higher anodic and cathodic peak currents, indicating improved electron transfer kinetics and an enhanced electroactive surface area. Among the investigated systems, the JUG–MWCNT/CPE electrode demonstrated the highest redox peak currents and the smallest peak-to-peak separation, suggesting the fastest heterogeneous electron transfer process and the most reversible electrochemical behavior. The incorporation of MWCNTs into the carbon paste matrix considerably improved the conductivity of the electrode, as evidenced by the reduction in *∆E_p_* from 355 mV for CPE to 210 mV for MWCNT/CPE. This improvement can be attributed to the excellent electrical conductivity and high specific surface area of the nanotubes, which promote faster electron transfer processes. Similarly, the JUG/CPE electrode also showed enhanced electrochemical activity compared to the unmodified electrode, reflected by increased peak currents and improved reversibility. However, the anodic-to-cathodic peak current ratio *I_pa_/I_pc_* of 2.8 suggests a less reversible electrochemical process than that of the remaining modified electrodes. Finally, the synergistic effect between JUG and MWCNTs in the hybrid electrode resulted in superior electrochemical properties, indicating the most favorable charge transfer kinetics among all tested systems. Moreover, the anodic-to-cathodic peak current ratio being close to unity (*I_pa_/I_pc_* = 1.1) confirms the quasi-reversible character of the electrochemical process occurring on the JUG-MWCNT/CPE surface.

The EIS measurements further confirmed the beneficial effect of electrode modification with the hybrid JUG-MWCNT mixture on interfacial electron transfer processes. The Nyquist plots shown in Figure 2B consist of depressed semicircles in the high-frequency region, followed by diffusion-controlled regions at lower frequencies, indicating the coexistence of charge transfer and diffusion phenomena. The bare CPE exhibited the highest charge transfer resistance (*R_ct_* = 40.5 kΩ), confirming relatively slow electron transfer kinetics at the electrode/electrolyte interface. The incorporation of MWCNTs into CPE resulted in an almost 5-fold decrease in the *R_ct_* value, owing to the formation of conductive pathways that facilitate charge transport. The JUG/CPE electrode also demonstrated a lower *R_ct_* value than the bare CPE, confirming the electroactive nature of juglone. Notably, the hybrid JUG–MWCNT/CPE electrode showed the lowest charge transfer resistance (2.2 kΩ), indicating the most efficient electron transfer process among the investigated electrodes. The improved electrochemical performance of the hybrid electrode is additionally supported by the calculated electroactive surface area (*A_el_*), which increased almost 6-times for JUG-MWCN/CPE compared to the unmodified CPE. The conductive nanotube network improves electron transport and increases the electroactive surface area, while juglone contributes additional electroactive sites. The significant reduction in the Warburg coefficient (σ) and the increase in the heterogeneous electron transfer rate constant (*k_s_*) for JUG-MWCNT/CPE indicate facilitated ion diffusion and accelerated electron transfer kinetics, highlighting the remarkable improvement in interfacial electrochemical properties achieved through the combined incorporation of juglone and MWCNTs.

The incorporation of juglone serves a pivotal role as an immobilized solid-state redox mediator within the carbon paste matrix. By leveraging its reversible quinone/hydroquinone transformation, juglone provides a low-energy pathway for electron transfer, effectively lowering overpotentials and significantly enhancing sensitivity compared to unmodified carbon platforms. Supported by the high-surface-area MWCNT network, this synergistic configuration maximizes the exposure of active redox centers, yielding excellent current amplification and long-term stability due to strong π–π confinement within the graphitic matrix. This specific chemical-to-structural alignment lowers activation energy barriers, providing a unique electronic microenvironment that drives the sensor’s competitive performance.

Further evidence of the superior electrochemical performance of the JUG-MWCNT/CPE electrode was provided by the analysis of the melatonin oxidation process recorded on the investigated electrodes. The DPV curves obtained for 0.2 mg L^−1^ MEL before baseline correction (Figure 2C) showed significant differences in the current response depending on the composition of the electrode. The bare CPE exhibited the lowest oxidation current over the entire potential range, indicating a limited electron transfer capability and poor electrocatalytic activity towards melatonin oxidation. In contrast, the modified electrodes generated substantially higher current responses, demonstrating the beneficial effect of both MWCNTs and juglone on the electrochemical performance of the sensor platform. This was especially apparent after background subtraction (Figure 2D), where the oxidation peak of melatonin became clearly distinguishable. For the bare CPE, only a weak oxidation peak was observed, whereas the introduction of MWCNT or JUG separately into the CPE resulted in a poorly shaped and non-symmetric peak, which makes MWCNT/CPE and JUG/CPE unsuitable for a reliable analysis of melatonin. In contrast, JUG-MWCNT/CPE allowed the recording of a stable and reproducible oxidation signal of MEL, which was approximately 11 times higher than that of the bare CPE. Furthermore, the significant shift in the MEL oxidation peak potential from +0.80 V on the bare CPE to +0.77 V on JUG-MWCNT/CPE indicates the electrocatalytic activity of the composite material. This behavior confirms that electrode modification facilitates the oxidation of MEL and enhances electron transfer kinetics by lowering the overpotential required for the oxidation process.

In summary, it can be concluded that the JUG-MWCNT/CPE electrode exhibits a favorable synergistic interaction between the juglone and the MWCNTs, resulting in enhanced electron transfer kinetics, improved charge transport, and increased accessibility of electroactive sites. These findings confirm that the hybrid modification significantly improves the analytical performance of the electrode for melatonin detection.

### 2.3. Electroanalysis of Melatonin on JUG-MWCNTs/CPE

#### 2.3.1. Investigation of the MEL Oxidation Mechanism

**Figure 3:**
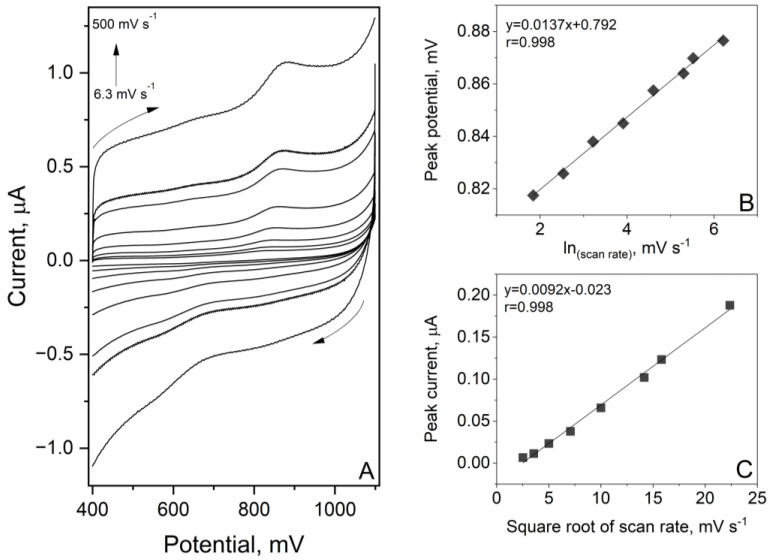
Cyclic voltammograms registered for 0.2 mg L^−1^ of melatonin in a 0.1 mol L^−1^ citric buffer (pH 3.0) in a scan rate range from 6.3 to 500 mV s^−1^ (**A**). MEL peak visible in the anodic scans. Dependences of the MEL peak potential on the natural logarithm of scan rate values (**B**) and on the square root of the scan rate (**C**).

The electrochemical response of MEL on JUG-MWCNTs/CPE was evaluated by examining how the supporting electrolyte’s pH and the scan rate (*v*) influence peak characteristics. As illustrated in Figure 3A, increasing the scan rate led to a concurrent rise in the anodic peak current (*I_pa_*) and a positive shift in the anodic peak potential (*E_pa_*), while the cathodic back-peak became increasingly negligible. A linear correlation was observed between the MEL peak current and the square root of the scan rate, yielding a slope of 0.0092 (*R^2^* = 0.998, Figure 3C). While such linearity often suggests a diffusion-limited process, the observed dependence of *E_pa_* on *v* strongly indicates that the reaction is governed by charge transfer kinetics. For processes under kinetic control, the relationship between the peak potential and scan rate is described by the following expression:

(4) $$E_{p}=K+\frac{RT}{2n\alpha F}\ln v$$

By substituting the universal constants (*F* = 96,485 C mol^−1^, *R* = 8.314 J (mol K)^−1^, and *T* = 298 K), the experimental slope for MEL oxidation was determined to be 0.0137 (Figure 3B). Under the assumption of a charge transfer coefficient (α) of 0.5, the calculated number of electrons (*n*) involved in the process was 1.87. This value confirms that the oxidation of MEL is a two-electron mechanism. This result was further verified using the following equation:

(5) nα=0.048Ep−Ep12

which reveals that two electrons were exchanged during MEL oxidation.

**Figure 4:**
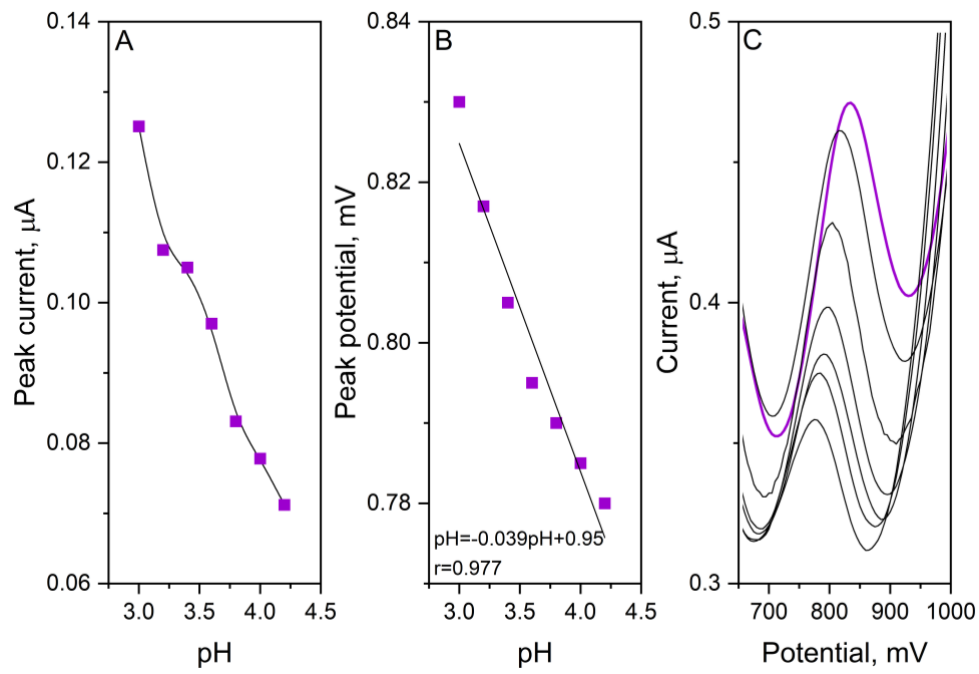
The dependence between the peak current (**A**) and potential (**B**) on the pH of the supporting electrolyte (**B**). Corresponding DPV voltammograms obtained for oxidation of 0.1 mg L^−1^ melatonin in 0.1 mol L^−1^ citric buffer of pH ranging from 3.0 to 4.2 (**C**). Violet line for a MEL voltammogram registered in chosen pH value. Other instrumental parameters are as in Point 3.4.

To fully investigate the MEL reaction mechanism on the JUG-MWCNTs/CP electrode, the impact of the pH value on the MEL oxidation peak was examined. For this purpose, DPVs for 0.1 mg L^−1^ MEL were recorded in 0.1 M citric buffer solutions with pH ranging from 3 to 4.2 (Figure 4C). The obtained curves exhibit a shift towards a less positive potential with the increasing supporting electrolyte pH value, which indicates that proton exchange accompanied electron transfer. The equation obtained from the MEL peak potential versus supporting electrolyte pH give a linear correlation in the form of

$$E_{p}=-0.039pH+0.95V$$

**Scheme 1:**
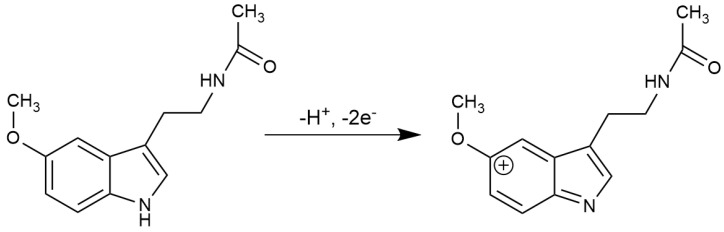
Possible mechanism of melatonin oxidation on JUG-MWCNTs/CPE.

In the studied case, the obtained slope suggests that the number of electrons exchanged during MEL oxidation is double that of the protons, which means that one proton and two electrons are involved in the MEL reaction on the JUG-MWCNTs/CPE surface. Considering these findings, the possible mechanism of the melatonin oxidation reaction on the JUG-MWCNTs/CP electrode is presented in Scheme 1. Graphite provides a stable structural and conductive bulk matrix; MWCNTs act as a high-surface-area conductive nano-scaffold that immobilizes the modifier via π–π interactions; and juglone functions as the electroactive mediator, facilitating fast interfacial electron transfer and signal amplification for the target analyte.

#### 2.3.2. Influence of DPV Parameters on Melatonin Peak

To maximize the detection sensitivity for melatonin at the JUG-MWCNTs/CP electrode, the parameters for differential pulse voltammetry (DPV) were systematically refined. The optimization involved evaluating a wide range of variables, specifically focusing on the step and pulse potentials alongside the waiting and sampling durations. Based on these evaluations, the following settings were identified as optimal for achieving peak performance: pulse potential (*dE*): 50 mV; step potential (*E_s_*): 5 mV; sampling time (*t_p_*): 10 ms; and waiting time (*t_w_*): 20 ms. These finalized conditions were consistently applied throughout all remaining experimental procedures.

#### 2.3.3. Interference Studies

To assess the selectivity of the JUG-MWCNT/CPE sensor, the impacts of various common biomolecules and pharmaceutical excipients on the electrochemical oxidation signal of MEL were investigated. Interference tests for glucose and caffeine involved introducing each substance at a concentration of 0.5 g L^−1^ into a supporting electrolyte containing 0.2 mg L^−1^ MEL, yielding an interferent-to-analyte ratio of 250:1. The potential interference of citric acid, uric acid, and ascorbic acid was similarly evaluated by incrementally spiking the 0.2 mg L^−1^ MEL solution with each compound up to a 100-fold excess. Additionally, the effects of solid excipients (starch, cellulose, titanium dioxide, and magnesium stearate) were assessed by adding 3 to 9 mg portions of the powdered materials directly into the voltammetric cell. The percentage deviation in the peak current relative to the pure MEL signal was calculated for each species. An interference effect was defined as a peak current variation exceeding ±10%, a noticeable peak shape distortion, or a shift in the peak potential.

Among the tested substances, significant deviations were caused only by glucose, resulting in a signal decrease of about 15%. Conversely, uric acid moderately suppressed the MEL response, reducing the peak current to 78% of its baseline value at a 25-fold excess. This decrease may stem from competitive adsorption on the electrode surface or partial fouling by uric acid oxidation products. Because the tablet excipients induced no observable interference, these findings validate the high selectivity of the fabricated sensor and its viability for reliable melatonin quantification in drugs and dietary supplements.

#### 2.3.4. Analytical Performance

**Figure 5:**
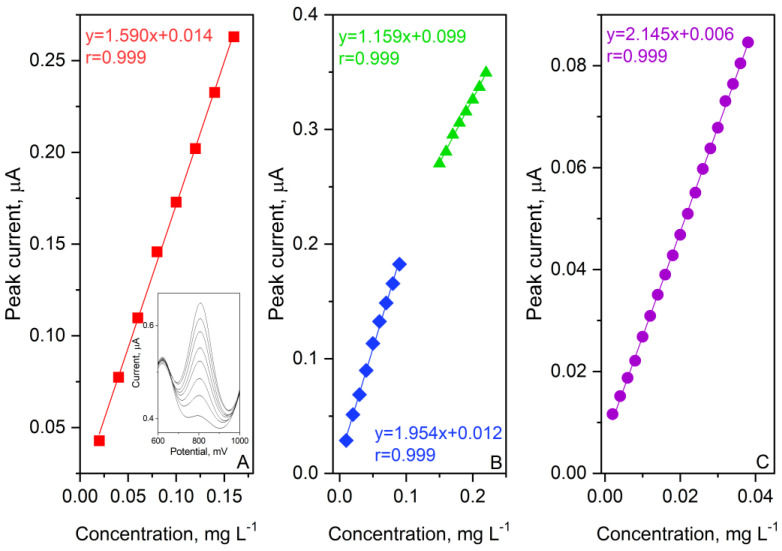
DPV melatonin calibration curves obtained for concentration ranges of (**A**) 0.02–0.16 mg L^−1^, (**B**) 0.01–0.09 mg L^−1^ and 0.15–0.22 mg L^−1^, and (**C**) 0.002–0.038 mg L^−1^ in 0.1 mol L^−1^ citric buffer (pH 3.0). Corresponding voltammograms are presented as an inset in (**A**). Other instrumental parameters are as in Point 3.4.

The analytical capabilities of the JUG-MWCNTs/CP electrode for melatonin quantification were evaluated using differential pulse voltammetry in a 0.1 mol L^−1^ citrate buffer (pH 3.0). Under optimized experimental parameters, four calibration curves were constructed using MEL standard additions of 0.02, 0.01, and 0.002 mg L^−1^. Each measurement was performed in triplicate, with the resulting mean peak currents utilized for the regression analysis depicted in Figure 5. As summarized in Table 3, the sensor demonstrated robust linearity across all investigated concentration ranges, with correlation coefficients (R^2^) ranging from 0.998 to 0.999. The calculated limit of detection (LOD) reached a minimum value of 0.49 µg L^−1^ (2.1·10^−9^ M). The precision of the proposed method was assessed via the relative standard deviation (RSD). An RSD of 1.02% was obtained for five successive measurements of a 0.02 mg L^−1^ MEL solution, indicating high signal stability. A comparison between independently prepared electrode surfaces yielded an RSD of 4.2%, confirming the reliability of the JUG-MWCNTs/CP fabrication process. The analytical performance of this modified paste electrode was benchmarked against that of previously reported voltammetric methods, with comparative data provided in Table 4. The JUG-MWCNTs/CP sensor demonstrated a competitive limit of detection (LOD) relative to previously reported electrochemical platforms.

A primary benefit of this specific architecture is the streamlined fabrication process, which utilizes a carbon paste method that bypasses the need for hazardous organic solvents. Longitudinal performance evaluations confirmed the electrode’s robust nature, showing consistent stability over a 30-day period and over approximately 1000 continuous voltammetric cycles. Between consecutive measurements, the electrode was stored in a sealed container under dry conditions at room temperature. The RSD value for the measured MEL signal between day 1 of the measurements and day 30 was about 4.2%. The superior electroanalytical sensitivity observed in this composite is likely due to the synergistic interaction between the high-surface-area multiwalled carbon nanotube scaffold and the juglone redox mediator. This multidimensional integration facilitates enhanced electron transfer kinetics compared to alternative electrode configurations. The fabrication of a composite paste electrode integrated with multiwalled carbon nanotubes and juglone yields a synergistic sensing platform, characterized by enhanced electron transfer kinetics and a high electroactive surface area. The MWCNT framework provides a robust conductive network that facilitates rapid charge transport, while the immobilization of juglone introduces a reversible redox mediator capable of undergoing proton-coupled electron transfer. This modification significantly reduces the overpotential for various analytes, exhibiting profound electrocatalytic activity and improved sensitivity compared to unmodified carbon paste electrodes. Furthermore, the π-π stacking interactions between the aromatic rings of juglone and the graphene walls of the MWCNTs ensure excellent chemical stability and reproducibility, making the electrode a promising candidate for high-performance electrochemical biosensors and environmental monitoring applications.

The practical utility of the proposed methodology was rigorously assessed through the quantification of melatonin within diverse commercial matrices, encompassing both regulated pharmaceutical formulations and over-the-counter dietary supplements. The inclusion of dietary supplements is particularly critical; unlike registered medicinal products, supplements often bypass stringent pre-market oversight, leading to documented concerns regarding ingredient purity and dosage accuracy. Consequently, robust analytical quality control is indispensable in the pharmaceutical industry to ensure consumer safety and therapeutic consistency.

Analytical samples were prepared following the extraction protocol detailed in the Tablets Section. To mitigate matrix interference—a common challenge in complex supplement formulations—all quantifications were executed via the standard addition method, utilizing three sequential fortifications of a certified MEL standard. To further validate the procedure’s accuracy, recovery studies were performed by spiking samples post-pretreatment. As summarized in Table 5, the recovery rates ranged from 96% to 104%, demonstrating that the developed procedure is highly effective for routine laboratory quality control and the detection of potential purity deviations in commercial products.

## 3. Materials and Methods

### 3.1. Chemicals and Solutions

The chemical reagents utilized in this study were of analytical grade and required no additional purification prior to use. Melatonin (98%) was sourced from Chemat (Gdańsk, Poland), while juglone (5-hydroxy-1,4-naphthoquinone, 97%) was obtained from AmBeed (Buffalo Grove, IL, USA). Both the synthetic graphite powder (particle size < 20 µm) and the functionalized multiwalled carbon nanotubes (L/D~1000, 99.9 wt.% MWCNT basis) were purchased from Sigma Aldrich (St. Louis, MO, USA). To prepare the stock solution of melatonin (1000 mg L^−1^), the reagent was weighed gravimetrically and dissolved in a 1:1 mixture of ethanol and double-distilled water. This stock was kept under refrigeration at 4 °C, and fresher, lower-concentration working standards were diluted from it on a daily basis. Additionally, a 0.1 mol L^−1^ citric buffer solution was formulated from citric acid and sodium citrate salt, both procured from Avantor (Gliwice, Poland).

### 3.2. Instrumentation

Voltammetric analyses were performed using an mtm-anko (Krakow, Poland) M161 multipurpose electrochemical analyzer coupled with an M164 electrode stand. Data collection and subsequent processing were handled through EALab (v. 2.1), Matlab R2026a, and Origin 2023b software. The experimental setup utilized a conventional three-electrode cell containing a platinum wire auxiliary electrode, an Ag/AgCl (3 mol L^−1^ KCl) reference electrode (Mineral, Poland), and a modified juglone/multiwalled carbon nanotube carbon paste electrode (JUG-MWCNT/CPE, 3 mm PEEK diameter, BASi, West Lafayette, IN, USA) as the working electrode.

Electrochemical impedance spectroscopy (EIS) investigations were conducted on a Metrohm Autolab system (Eco Chemie AUT32N.FRA2, Herisau, Switzerland) operated via NOVA 2.1 software. This cell configuration consisted of a platinum rod counter electrode, an Ag./AgCl/KCl (3 mol L^−1^) reference, and the modified paste working electrodes. EIS measurements were conducted in a solution containing 1 mmol L^−1^ K_3_ [Fe(CN)_6_] and 1 mol L^−1^ KCl. Spectra were recorded at the formal potential of the redox probe, applying a 50 mV sinusoidal amplitude over a frequency spectrum spanning from 100 kHz down to 10 mHz. During both voltammetric and impedance characterizations, the solutions were agitated at approximately 200 rpm using a Teflon^®^-coated magnetic stir bar (Kartell, Italy). For experiments executed in the negative potential region, solutions were initially deoxygenated by purging with argon gas. A SevenCompact S210 pH meter (Mettler Toledo, Greifensee, Switzerland) was used to adjust the pH of the citric buffer.

Surface morphology and imaging were evaluated using an Apreo 2 S LoVac ultra-high-resolution scanning electron microscope (Thermo Fisher Scientific, Breda, The Netherlands), integrated with an Octane Elect X-ray microanalysis system (EDAX Ametek GmbH, Tilburg, The Netherlands), alongside a Phenom ProX system (Thermo Fisher Scientific, Breda, The Netherlands). Imaging was carried out under low-vacuum conditions utilizing an accelerating voltage between 10 and 18 kV, with signals captured via secondary electron (SE) and backscattered electron (BSE) detectors.

The textural properties of the electrode modifiers were determined by nitrogen adsorption–desorption measurements at 77 K using an ASAP 2010 analyzer (Micromeritics Instruments, Norcross, GA, USA). To remove adsorbed impurities and moisture, the juglone sample was degassed at 393 K, while graphite and MWCNT were degassed at 623 K for 24 h before analysis. The specific surface area was calculated according to the Brunauer–Emmett–Teller (BET) method, while the pore size distribution and porosity parameters were evaluated using the Barrett–Joyner–Halenda (BJH) model. The infrared spectrum of juglone in the mid-infrared (MIR) range (4000–400 cm^−1^) was recorded using a Bruker Vertex 70v FTIR spectrometer software spectrometer (Bruker, Billerica, MA, USA). Measurements were carried out using the standard KBr pellet technique, collecting 256 scans at a resolution of 4 cm^−1^. The Raman spectra of graphite and MWCNT were acquired with a WITec Alpha 300 M+ Raman system software system. The instrument was equipped with a 600 grooves mm^−1^ grating and a 532 nm laser, with the laser power adjusted to avoid sample damage. A Zeiss Epiplan Neofluar 20× long-working-distance objective was used, providing a laser spot size of approximately 0.5 μm. Raman mapping was performed on 40 μm × 40 μm areas with a step size of 0.5 μm, and each spectrum was collected with an integration time of 0.5 s. Data processing was carried out using WITec Project FIVE 5.3 PRO software.

### 3.3. Paste Electrode Preparation

**Figure 6:**
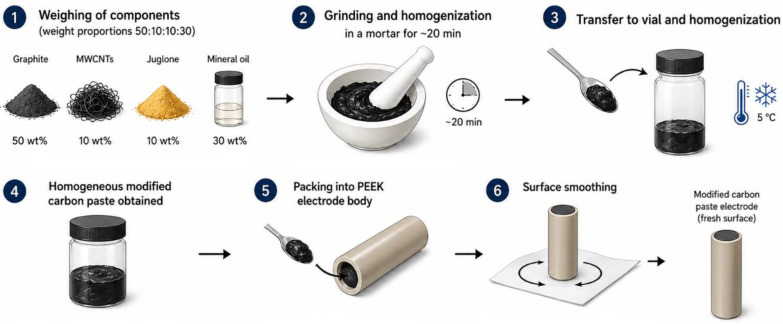
Visual presentation of JUG-MWCNTs/CP electrode preparation.

The paste constituting the core of the working electrode consisted of graphite, multiwalled carbon nanotubes, juglone, and mineral oil in the weight proportions of 50:10:10:30. After weighting, the powders with mineral oil were thoroughly homogenized by grinding in a mortar for approximately 20 min. The obtained paste was transferred to a vial and left to homogenize for 48 h in the fridge (5 °C). The prepared homogenous modified carbon paste was firmly packed into the cavity of the PEEK electrode body, and the surface was smoothed on clean paper to obtain a fresh, reproducible electroactive surface. Figure 6 illustrates the electrode preparation process.

### 3.4. Voltammetric Procedures

The electrochemical properties of the CPE and modified CPEs were evaluated using cyclic voltammetry (CV) and electrochemical impedance spectroscopy (EIS) in a 0.1 mol L^−1^ KCl electrolyte containing 2 mmol L^−1^ of the reversible redox probe [Fe(CN)_6_]^4−^. For EIS measurements, a sinusoidal signal with a frequency ranging from 100 kHz to 25 mHz and 10 mV amplitude superimposed on the formal potential of K_4_[Fe(CN)_6_] was applied. The resulting impedance spectra were analyzed by fitting them to an equivalent electrical circuit (EEC) model in order to extract the corresponding electrochemical parameters. The electrochemical behavior of melatonin (MEL) at JUG-MWCNTs/CPE was investigated using both cyclic voltammetry (CV) and differential pulse voltammetry (DPV). CV was performed to elucidate the underlying mechanisms and clarify the redox kinetics of the melatonin oxidation pathway on the modified electrode. Conversely, DPV was utilized as the primary method for high-sensitivity quantitative determination.

All voltammograms were collected in a citric buffer solution adjusted to pH 3.0, sweeping across a potential range of −400 to 1100 mV. The fine-tuned parameters for the DPV measurements consisted of a step potential (E_s_) of 5 mV, a pulse amplitude (dE) of 50 mV, a waiting time (t_w_) of 20 ms, and a sampling time (t_p_) of 10 ms. For the mechanistic study of MEL oxidation via CV, the scan rates were systematically altered between 6.3 and 500 mV s^−1^.

To guarantee the precision of the quantitative data, baseline corrections were applied to the raw voltammograms using a polynomial fitting method. This allowed the background charging current to be mathematically modeled and deducted from the total measured current, effectively isolating the Faradaic signal of the analyte.

### 3.5. Sample Preparation

#### Tablets

For the quantitative evaluation of melatonin in commercial products, one pharmaceutical brand and two distinct dietary supplements were procured from a local pharmacy. To ensure uniformity, three tablets from each brand were thoroughly pulverized and homogenized using a mortar and pestle. Accurately weighed portions of the resulting powders were then transferred to volumetric flasks and dissolved in a mixture of ethanol and double-distilled water to completely solubilize the melatonin and achieve the targeted stock concentrations.

Prior to electrochemical analysis, the prepared solutions were passed through regenerated cellulose (RC) syringe filters (Biosens, Warszawa, Poland) featuring a 0.45 µm pore diameter to remove any insoluble excipients. The resulting filtrates were collected in Eppendorf tubes and appropriately diluted with double-distilled water to reach the required working concentrations.

## 4. Conclusions

In this work, a multiwalled carbon nanotube/carbon paste electrode modified with juglone (JUG-MWCNT/CPE) was engineered and evaluated for the electrochemical detection of melatonin in pharmaceutical and supplement forms. By combining the quinone properties of juglone with functionalized multiwalled carbon nanotubes, the sensor achieved superior electron transfer kinetics and enhanced catalytic activity, leading to heightened sensitivity and selectivity for the analyte. The platform is characterized by a wide linear response and a nanomolar limit of detection, functioning effectively without the need for preconcentration. Due to its cost-efficient fabrication, long-term stability, and reliable performance in complex matrices, this electrode offers a practical solution for routine quality control in drug analysis. While the developed JUG–MWCNT/CPE sensor offers competitive sensitivity and a low detection limit, certain limitations remain to be addressed in future work. As is characteristic of carbon paste matrices, long-term continuous immersion can lead to gradual surface fouling or binder-dependent mechanical degradation. Therefore, future research will focus on incorporating polymer-protective membranes to enhance mechanical stability and fouling resistance. Additionally, we aim to adapt this synergistic material into screen-printed electrode formats to facilitate fully automated, disposable, and on-site field analysis.

## Figures and Tables

**Table 1:** Textural parameters of employed materials.

Material	Surface Area, m^2^ g^−1^			Pore Volume, cm^3^ g^−1^		Average Pore Diameter, nm
	*S_BET_*	*S_micro_*	*S_ext_*	*V_micro+mezo_*	*V_micro_*	
Graphite	11.28	–	11.13	0.04	–	1.25
MWCNT	290.56	46.06	244.50	0.09	0.02	1.24
Juglone	19.07	–	19.06	0.002	–	0.48

*S_BET_*—BET surface area; *S_micro_*—micropore area; *S_ext_*—external surface area; *V_micro+mezo_*—micropore + mesopore volume; *V_micro_*—micropore volume; dash–not calculated.

**Table 2:** Comparison of electrochemical properties of tested electrodes determined via CV and EIS measurements.

Parameter	Unit	Working Electrode			
		CPE	MWCNT/CPE	JUG/CPE	JUG-MWCNT/CPE
Formal potential, *E_f_*	mV	210	200	192	186
Peak separation, *∆E*	mV	355	210	384	161
Anodic peak current, *I_pa_*	µA	6.1	22.3	17.7	32.3
Anodic-to-cathodic peak current, *I_pa_/I_pc_*	-	1.3	1.2	2.8	1.1
Charge transfer resistance, *R_ct_*	kΩ	40.5	8.6	14.6	2.2
Warburg coefficient, *σ*	kΩs^−1/2^	6.2	1.3	1.9	1.2
Electroactive surface area, *A_el_*	mm^2^	2.3	10.6	7.6	12.1
Double-layer capacitance, *C_dl_*	µF cm^−2^	5.3	3.9	3.3	3.8
Heterogeneous rate constant, *k_s_*	m s^−1^	2.9 × 10^−6^	2.9 × 10^−6^	2.4 × 10^−6^	1.0 × 10^−5^

**Table 3:** Analytical parameters obtained for the developed procedures for melatonin determination.

Parameter	Linear Range	Slope	Intercept	R	LOD
Unit	mg L^−1^	µA L mg^−1^	µA	-	µg L^−1^ (nM)
Figure 5A	0.02–0.16	1.590 ± 0.022	0.014 ± 0.002	0.999	4.15 (17.9)
Figure 5B	0.01–0.09	1.954 ± 0.039	0.012 ± 0.002	0.999	3.37 (14.5)
	0.15–0.22	1.159 ± 0.021	0.099 ± 0.004	0.998	10.0 (43.1)
Figure 5C	0.002–0.038	2.145 ± 0.015	0.0056 ± 0.00035	0.999	0.49 (2.10)

**Table 4:** Comparison of the melatonin voltametric determination methods.

Electrode	Linear Range, µM	Limit of Detection, nM	Ref.
SnO_2_-Co_3_O_4_@rGO	0.02–6	4.1	[48]
CBGC	0.0028–12	19	[49]
Au-MoS_2_	0.033–10	15.7	[50]
SNGCE-AuNPs	0.02–0.3	8.4	[51]
Gr-Fe_3_O_4_/CPE	0.02–5.80	8.4	[52]
GCE/MagNPs/Cdots	0.05–13.5	4.4	[53]
ZnFe_2_O_4_ NPs	6.5–145	3000	[54]
Gr-Av	10–100	47	[55]
PdNP@Al_2_O/CPE	0.006–1400	21.6	[56]
Tween/h-BDD/SPE	0.057–10 10–200	17	[57]
CPE	0.5–80	83	[58]
GCE/3DG/Au NPs	0.05−50	8.2	[59]
GCE/3DG/Au NPs	0.05−50	8.2	[59]
SPE	0.25–75	25.8	[60]
GCE/f-MWCNT-AuNP-Si4Pic^+^Cl^−^	4.9–55.5	1600	[61]
RGO-Cu_0.5_Co_0.5_Fe_2_O_4_/Pt	0.2–20	172	[62]
MIP-PTBO/ MWCNTs/GCE	1–1000	27	[63]
MIPs/4-AMP/PEDOT/GCE	0–100	171	[64]
e-CS/MWCNTs/GCE	20–1000	22,000	[65]
JUG-MWCTs/CPE	0.002–0.16	2.1	This work

SnO_2_-Co_3_O_4_@rGO—ionic liquid carbon paste electrode modified with reduced graphene oxides decorated with SnO_2_-Co_3_O_4_; CBGC—carbon black modified glassy carbon electrode; Au-MoS_2_—self-assembled Au nanoparticle–MoS_2_ nanoflake; SNGCE-AuNPs—sonogel–carbon electrode material and Au nanoparticles; Gr-Fe_3_O_4_/CPE—graphene decorated with Fe_3_O_4_ magnetic nanoparticles on a carbon paste electrode; GCE/MagNPs/Cdots—GCE coated with Fe_3_O_4_ nanoparticles and carbon quantum dots; ZnFe_2_O_4_ NPs—modified carbon paste electrode with zinc ferrite nanoparticles; PdNP@Al_2_O/CPE—carbon paste electrode with Al_2_O_3_-supported palladium nanoparticles; Tween/h-BDD/SPE—Tween-coated h-BDD modified screen-printed electrode; CPE—carbon paste electrode; GCE/3DG/Au NPs—glassy carbon electrode modified with unfunctionalized macroporous graphene networks decorated with gold nanoparticles; SPE—screen-printed electrode; GCE/f-MWCNT-AuNP-Si4Pic^+^Cl^−^—electrochemical platform modified with gold nanoparticles stabilized by 3-n-propyl-(4-methylpyridinium) silsesquioxane chloride and functionalized multiwalled carbon nanotubes; RGO-Cu_0.5_Co_0.5_Fe_2_O_4_/Pt—reduced graphene oxide-Cu_0.5_Co_0.5_Fe_2_O_4_ modified platinum electrode; MIP-PTBO/MWCNTs/GCE—molecularly imprinted polymer with polytoluidine blue O and multiwalled carbon nanotubes on a glassy carbon electrode; MIPs/4-AMP/PEDOT/GCE—4-aminothiophenol, 3,4-ethylenedioxythiophene modified glassy carbon electrode; e-CS/MWCNTs/GCE—glassy carbon electrode modified with a composite consisting of electrodeposited chitosan and carboxylated multiwalled carbon nanotubes.

**Table 5:** Results of melatonin determination in drug and dietary supplements.

Sample	MEL Declared, mg	MEL Found, mg	Recovery, %
Drug	3.00	3.04	101
Dietary supplement 1	1.00	0.96	96
Dietary supplement 2	1.00	1.04	104

## Data Availability

The data sets used in this study are available from the corresponding author upon reasonable request.
